# Brain Morphological Alterations in Adults with Spinal Muscular Atrophy Types 2 and 3: A CAT12-Derived Region-Based and Surface-Based Morphometry Study

**DOI:** 10.3390/jcm15145666

**Published:** 2026-07-20

**Authors:** Aleksandra Rubin-Starczewska, Przemysław Podgórski, Jakub Ubysz, Magdalena Koszewicz, Jagoda Jacków-Nowicka, Oliwia Kieczka, Stylianos Kapetanakis, Grzegorz Trybek, Joanna Bladowska

**Affiliations:** 1 Division of General and Interventional Radiology and Neuroradiology, Department of Radiology, University Clinical Hospital in Wroclaw, 50-556 Wroclaw, Poland; aleksandra.rubin@gmail.com (A.R.-S.); przemyslaw.podgorski@umw.edu.pl (P.P.); 2 Division of General and Interventional Radiology and Neuroradiology, Department of Radiology, Faculty of Medicine, Wroclaw Medical University, 50-367 Wroclaw, Poland; 3 Clinical Department of Neurology, University Centre of Neurology and Neurosurgery, Faculty of Medicine, Wroclaw Medical University, 50-367 Wroclaw, Poland; ubysz.jakub@gmail.com; 4 Clinical Neurophysiology Laboratory, University Centre of Neurology and Neurosurgery, Faculty of Medicine, Wroclaw Medical University, 50-367 Wroclaw, Poland; magdalena.koszewicz@umw.edu.pl; 5 Department of Preclinical Sciences, Pharmacology and Medical Diagnostics, Faculty of Medicine, Wroclaw University of Science and Technology, 50-370 Wroclaw, Poland; 6 Department of Radiology, 4th Military Clinical Hospital with Polyclinic in Wroclaw, 50-981 Wroclaw, Poland; 7 Faculty of Medicine, Wroclaw Medical University, 50-367 Wroclaw, Poland; 8 Spine Department and Deformities, Interbalkan European Medical Center, Asklipiou 10, 555 35 Pilea Chortiatis, Thessaloniki, Greece; stkapetanakis@yahoo.gr; 9 Department of Oral Surgery, Pomeranian Medical University in Szczecin, 70-111 Szczecin, Poland; 10 Department of Maxillofacial Surgery, 4th Military Clinical Hospital with Polyclinic in Wroclaw, 50-981 Wroclaw, Poland; 11 Department of Radiology, T. Marciniak Lower Silesian Specialist Hospital, Emergency Medicine Center, 54-049 Wroclaw, Poland

**Keywords:** spinal muscular atrophy, SMA, voxel-based morphometry, region-based morphometry, surface-based morphometry, magnetic resonance imaging, thalamus, cortical thickness, sulcal depth, neurodegeneration

## Abstract

**Background/Objectives:** Spinal muscular atrophy (SMA) types 2 and 3 are increasingly recognised as potentially multisystem disorders that may involve the central nervous system. However, the extent and pattern of brain structural alterations in adults remain insufficiently characterised. This study aimed to assess brain morphology in adult patients with SMA using Computational Anatomy Toolbox 12 (CAT12)-derived region-based morphometry and surface-based morphometry. **Methods:** The study included 27 right-handed adult patients with SMA types 2 and 3 and 27 age- and sex-matched healthy controls. MRI examinations were performed before initiation of disease-modifying therapy or during the early loading phase of intrathecal nusinersen treatment, with a maximum exposure of two months and no more than three doses before MRI. The groups did not differ significantly in age, sex distribution, or total intracranial volume. Regional volumetric measures and cortical surface-based parameters were extracted from predefined atlas-based regions of interest. Between-group differences and associations with selected clinical and genetic variables were analysed. *p*-values were corrected for multiple comparisons using the Benjamini–Hochberg false discovery rate procedure. **Results:** CAT12-derived region-based morphometry showed a significant reduction in grey matter volume in the left thalamus in patients with SMA compared with healthy controls. Surface-based morphometry revealed increased sulcal depth in the left orbital sulcus as the only cortical finding that remained significant after correction for multiple comparisons. Additional differences in cortical thickness and sulcal depth were observed only at nominal or exploratory thresholds and did not survive correction for multiple comparisons. **Conclusions:** Adults with SMA types 2 and 3 showed selected brain structural alterations, particularly reduced grey matter volume in the left thalamus and increased sulcal depth in the left orbital sulcus. These findings are consistent with the concept that SMA may extend beyond lower motor neuron degeneration. Larger longitudinal multimodal studies are warranted to validate these observations and clarify their clinical significance.

## 1. Introduction

Spinal muscular atrophy (SMA) is a rare, progressive genetic neuromuscular disorder caused by pathogenic variants in the SMN1 gene on chromosome 5q, most commonly homozygous deletion of exon 7, resulting in reduced levels of survival motor neuron (SMN) protein [1,2,3]. SMN deficiency leads primarily to degeneration of lower motor neurons in the anterior horns of the spinal cord and motor nuclei of the brainstem [4,5], clinically manifesting as progressive muscle weakness, atrophy, disability, and, in severe forms, early death [6]. Scoliosis is a common complication of the disease [7]. The clinical spectrum of SMA ranges from severe early-onset forms to milder later-onset phenotypes [3,4,8]. The SMN2 gene may partially compensate for SMN1 loss; however, most SMN2 transcripts produce a truncated and less functional SMN protein, and disease severity is partly modified by SMN2 copy number [9,10,11,12].

SMA is traditionally classified into types 0–4 according to age at symptom onset and the highest motor milestone achieved [3,13,14,15]. Although SMA is primarily considered a lower motor neuron disease, multisystem involvement has also been reported, including abnormalities affecting the heart, kidneys, liver, pancreas, bones, and immune system [16,17]. Neuropathological studies in humans and animal models have demonstrated central nervous system involvement, particularly in severe SMA phenotypes, with reported abnormalities in the thalamus, basal ganglia, cranial nerve nuclei, motor cortex, and cerebellum [18,19,20]. Neuroimaging studies have also shown brain atrophy and involvement of the thalamus and basal ganglia in severe SMA forms, especially types 0 and 1 [18], whereas more subtle or regionally limited brain alterations have been described in milder SMA types 3 and 4 [21,22]. Despite these observations, the extent and clinical relevance of central nervous system involvement in SMA remain incompletely understood.

Volumetric MRI techniques, including voxel-based morphometry (VBM) and surface-based morphometry (SBM), allow quantitative assessment of structural brain alterations. VBM is an automated method used to assess local differences in grey and white matter volume or concentration after tissue segmentation and spatial normalisation, enabling group-level comparisons of brain morphology [19,20]. However, voxel-wise morphometric findings do not always correspond directly to discrete anatomical structures and may depend on preprocessing and statistical inference procedures [21].

SBM provides complementary information by analysing cortical surface morphology. It enables assessment of parameters such as cortical thickness, gyrification index, sulcal depth, and fractal dimension. These measures may help detect subtle and spatially localised alterations in cortical architecture associated with neurodevelopmental, neurodegenerative, or disease-related processes [22].

Advanced structural MRI morphometric approaches, including CAT12-derived region-based morphometry and surface-based morphometry, may therefore help identify brain alterations in SMA and assess whether in vivo imaging findings are consistent with previously reported neuropathological observations. However, associations between imaging findings and clinical, genetic, or functional parameters should be interpreted cautiously, particularly in small and clinically heterogeneous cohorts.

### Study Objectives

This study aimed to evaluate brain morphology in adults with SMA types 2 and 3 using CAT12-derived region-based morphometry and surface-based morphometry. The objectives were to identify structural brain differences between patients with SMA and healthy controls and to explore potential associations between morphometric findings and selected clinical and genetic variables.

## 2. Methods

MRI examinations were conducted at a tertiary academic centre between October 2021 and March 2023. The study included 27 patients with SMA types 2, 3a, and 3b and 27 age- and sex-matched healthy controls. The study was approved by the local institutional bioethics committee (approval No. KB-70/2021), and all participants provided informed consent.

### 2.1. Study Group

The SMA group included 27 right-handed adults with genetically confirmed 5q spinal muscular atrophy: 15 females and 12 males, aged 23–60 years, with a mean age of 39.8 years. Four participants had SMA type 2, and 23 had SMA type 3, including 12 patients with type 3a and 11 patients with type 3b. SMN2 copy number was assessed in all patients (Table 1).

At the time of MRI acquisition, patients were either undergoing eligibility assessment for disease-modifying therapy or were in the initial loading phase of intrathecal nusinersen treatment. The maximum duration of nusinersen exposure before MRI was two months, corresponding to no more than three administered doses.

Motor function was assessed in all patients using the Hammersmith Functional Motor Scale Expanded (HFMSE). Non-ambulatory patients were additionally evaluated using the Children’s Hospital of Philadelphia Infant Test of Neuromuscular Disorders (CHOP-INTEND).

Cognitive function was screened using the Montreal Cognitive Assessment (MoCA) to exclude significant cognitive impairment. All patients scored 26 points or higher. Detailed neuropsychological assessment was beyond the scope of the present study.

Inclusion criteria for the SMA group were age over 18 years, genetically confirmed SMA diagnosis, and written informed consent. Exclusion criteria included claustrophobia, severe body deformities preventing MRI acquisition, active infection, other neurological disorders, history of head trauma, and MR-incompatible implants.

### 2.2. Control Group

The control group included 27 right-handed healthy volunteers: 15 females and 12 males, aged 27–61 years, with a mean age of 39.7 years. All controls provided informed consent. Eligibility criteria included age over 18 years and no history of chronic disease, central nervous system injury, or neurological disorder. Exclusion criteria were the same as for the SMA group.

### 2.3. MRI Acquisition

MRI examinations in both groups were performed using a 3T Philips Ingenia Best Netherlands with a 32-channel head coil. The imaging protocol included T1- and T2-weighted sequences in axial, sagittal, and coronal planes, axial 3D FLAIR, diffusion-weighted imaging, and susceptibility-weighted imaging.

The 3D T1-weighted sequence used for morphometric analysis was acquired with the following parameters: TE/TR, 4/8 ms; flip angle, 8°; one excitation; in-plane resolution, 1 mm × 1 mm; effective slice thickness, 1 mm; 170 slices without interslice gaps; matrix, 250 mm × 250 mm; and field of view, 25 cm × 25 cm. Volumetric 3D T1-weighted images were used for subsequent brain morphometric analyses.

### 2.4. Region-Based Morphometry

Structural 3D T1-weighted MR images were converted from DICOM to NIfTI format using MRIcron and processed with SPM12 and the Computational Anatomy Toolbox (CAT12). The CAT12 preprocessing pipeline included correction for intensity inhomogeneity, affine registration, nonlinear spatial normalisation, tissue segmentation, and estimation of total intracranial volume.

T1-weighted images were segmented into grey matter, white matter, and cerebrospinal fluid compartments. Spatial normalisation to MNI space was performed within the CAT12/SPM12 framework. Modulated tissue maps were generated to preserve regional volumetric information after spatial normalisation. CAT12 was also used to estimate global tissue volumes, including total grey matter volume, total white matter volume, cerebrospinal fluid volume, and total intracranial volume.

For region-based morphometric analysis, CAT12-derived volumetric values were extracted from predefined atlas-based anatomical regions of interest. The final statistical analysis was performed on exported regional CAT12-derived values rather than on voxel-wise statistical maps. Therefore, the reported findings represent a CAT12-derived region-based volumetric analysis. Voxel-wise peak MNI coordinates, cluster extent, peak voxel statistics, voxel-level inference, cluster-level inference, and voxel- or cluster-level family-wise error correction were not applied.

### 2.5. Surface-Based Morphometry

Surface-based morphometric processing was performed using CAT12 implemented in SPM12. The preprocessing pipeline included noise reduction using Spatially Adaptive Non-Local Means filtering, correction of intensity inhomogeneity and image artefacts, affine registration, tissue segmentation, skull stripping, and brain extraction.

Structural images were segmented into grey matter, white matter, and cerebrospinal fluid compartments. CAT12 volumetric preprocessing included adaptive segmentation, partial volume estimation, and spatial normalisation to MNI space. These preprocessing steps provided the basis for subsequent cortical surface reconstruction and surface-based morphometric analysis.

Cortical surface reconstruction and estimation of surface-derived parameters were performed using the CAT12 surface-based processing pipeline. The analysed cortical metrics included cortical thickness, sulcal depth, gyrification index, and fractal dimension. Regional surface-based parameters were extracted from atlas-defined cortical regions of interest using the Destrieux atlas, also known as the aparc.a2009s parcellation.

Cortical thickness, sulcal depth, gyrification index, and fractal dimension were evaluated across 150 cortical regions defined by the Destrieux atlas as implemented in CAT12. Quality control was performed using CAT12 preprocessing and postprocessing quality assessment tools, including visual inspection of segmentation, spatial normalisation, and cortical surface reconstruction outputs. Final image quality ratings ranged from 84% to 96% according to the CAT12 quality assessment.

### 2.6. Statistical Analysis

The statistical analysis included two cohorts of 27 participants each. Region-based volumetric measures and surface-based morphometric parameters were analysed using exported CAT12-derived regional values. Surface-based parameters were assessed across 150 cortical regions defined by the Destrieux atlas.

The SMA and control groups were matched at the cohort level and did not differ significantly in age, sex distribution, or total intracranial volume. Therefore, between-group comparisons of regional CAT12-derived morphometric measures were performed without additional covariate adjustment for age, sex, or total intracranial volume.

Continuous variables were summarised as means and standard deviations for approximately normally distributed data, or as medians and interquartile ranges for non-normally distributed data. Normality was assessed using the Shapiro–Wilk test and additionally evaluated using skewness and kurtosis. Homogeneity of variance was assessed using Levene’s test.

Between-group comparisons of regional morphometric parameters were performed using independent-samples tests. Student’s *t*-test was used for normally distributed variables with homogeneous variances, Welch’s *t*-test for normally distributed variables with unequal variances, and the Mann–Whitney U test for variables that did not meet the criteria for normality. Due to the number of regional comparisons, *p*-values were adjusted for multiple testing using the Benjamini–Hochberg false discovery rate procedure within the respective family of regional tests. Statistical significance was defined as an FDR-adjusted *p*-value below 0.05.

Results were reported as effect estimates, 95% confidence intervals, uncorrected *p*-values, and FDR-adjusted *p*-values. Final statistical analyses were performed in R.

Associations between morphometric parameters and selected clinical or genetic variables were explored within the SMA group. Depending on the type and distribution of the analysed variables, correlation analyses, independent-samples tests, analysis of variance, or non-parametric alternatives were applied as appropriate. These analyses were considered exploratory and were interpreted descriptively, with effect estimates and 95% confidence intervals reported where applicable.

## 3. Results

### 3.1. Region-Based Morphometry

CAT12-derived region-based morphometry revealed a significant reduction in grey matter volume in the left thalamus in patients with SMA compared with healthy controls. This analysis was based on exported atlas-defined regional volumetric values rather than on whole-brain voxel-wise statistical maps. The between-group difference in the left thalamic grey matter volume remained statistically significant after correction for multiple comparisons using the Benjamini–Hochberg false discovery rate procedure (FDR-adjusted *p* < 0.001).

### 3.2. Surface-Based Morphometry

#### 3.2.1. Sulcal Depth

Surface-based morphometry revealed a significant between-group difference in sulcal depth in the left orbital sulcus. In this region, patients with SMA showed more than 1 mm greater sulcal depth than healthy controls. This difference remained statistically significant after correction for multiple comparisons (uncorrected *p* < 0.001; FDR-adjusted *p* = 0.012). No other cortical region remained significant after FDR correction.

Several additional regions showed nominal between-group differences before correction for multiple comparisons, but these findings did not survive FDR adjustment. The left straight gyrus and right orbital sulcus showed uncorrected *p*-values of 0.003 and 0.005, respectively, with corresponding FDR-adjusted *p*-values of 0.225 and 0.226. Compared with healthy controls, patients with SMA showed 0.47 mm lower sulcal depth in the left straight gyrus and 0.66 mm greater sulcal depth in the right orbital sulcus (Table 2).

Additional exploratory nominal differences in sulcal depth were observed in the right middle frontal gyrus, right inferior temporal gyrus, right suborbital sulcus, and left inferior temporal sulcus. In these regions, differences ranged from 0.36 mm greater to 0.76 mm lower sulcal depth in the SMA group compared with controls; however, none of these findings remained significant after FDR correction.

Increased sulcal depth of the left orbital sulcus was therefore the only surface-based morphometric finding that survived correction for multiple comparisons. The remaining cortical differences should be interpreted as exploratory observations. Although these nominal findings may suggest subtle cortical morphological alterations in SMA, confirmation in larger independent cohorts is required before their biological significance can be inferred.

#### 3.2.2. Cortical Thickness

No regional differences in cortical thickness between the SMA and control groups remained statistically significant after correction for multiple comparisons. The lowest FDR-adjusted *p*-values were observed in the left occipital pole and left cuneus. These regions showed nominal between-group differences before correction, with uncorrected *p*-values of <0.001 and 0.001, respectively, and corresponding FDR-adjusted *p*-values of 0.065 and 0.082. In both regions, the SMA group showed approximately 0.10 mm greater cortical thickness than the control group. As these findings did not meet the predefined threshold for statistical significance after FDR correction, they should be interpreted as exploratory observations.

#### 3.2.3. Other Surface-Based Parameters

For fractal dimension and gyrification index, no regional differences between the SMA and control groups remained statistically significant after correction for multiple comparisons. No region showed an FDR-adjusted *p*-value below the predefined significance threshold of 0.05.

### 3.3. Exploratory Associations with Clinical and Genetic Variables

Exploratory analyses were performed to assess whether selected surface-based morphometric parameters were associated with demographic, clinical, or genetic variables within the SMA group.

Sex-related differences were observed in the sulcal depth of the left and right orbital sulci. Female patients showed lower sulcal depth in these regions than male patients, with mean differences of −1.12 mm and −1.08 mm, respectively (MD = −1.12, *p* = 0.001; MD = −1.08, *p* = 0.001).

Age-related differences were also observed. In patients younger than 40 years, higher values of selected surface-based parameters were found in the right orbital sulcus, right inferior temporal gyrus, and left cuneus, with mean differences of 0.96, 0.55, and 0.13, respectively (*p* = 0.011, *p* = 0.046, and *p* = 0.009).

Exploratory subgroup analyses according to SMN2 copy number suggested differences between healthy controls and SMA subgroups with three or four SMN2 copies. Differences in the sulcal depth of the left orbital sulcus were observed in relation to both SMN2 copy-number subgroups. Additional differences were noted in the right orbital sulcus, right middle frontal gyrus, left straight gyrus, left occipital pole, and left cuneus. Because of the limited subgroup sizes, these observations should be interpreted as hypothesis-generating rather than as definitive genotype–phenotype associations.

Exploratory analyses according to SMA subtype suggested greater sulcal depth of the left orbital sulcus in SMA subgroups compared with controls. In the SMA type 3a subgroup, additional differences in cortical thickness were observed in the left occipital pole and left cuneus compared with controls. Given the small number of patients with SMA type 2, subtype-related findings should be interpreted with caution and require confirmation in larger cohorts.

Analyses stratified by HFMSE score suggested differences in selected brain surface-based parameters between controls and SMA subgroups. Both HFMSE subgroups, defined as HFMSE ≤ 10 and HFMSE > 10, differed from controls in the left orbital sulcus and occipital pole. The HFMSE ≤ 10 subgroup also showed differences in the right orbital sulcus, left inferior temporal sulcus, right inferior temporal gyrus, and left cuneus, whereas the HFMSE > 10 subgroup showed differences in the left straight gyrus compared with controls. These findings reflect differences between controls and selected SMA subgroups and should not be interpreted as robust within-SMA correlations with clinical severity.

No significant associations were found between the analysed radiological parameters and the presence of scoliosis or ambulatory status, with all *p*-values greater than 0.05.

Overall, subgroup comparisons according to SMN2 copy number, SMA subtype, and HFMSE category showed significant differences only between controls and selected SMA subgroups. These results suggest an association between group membership and selected morphometric values, but they do not demonstrate a consistent relationship between morphometric parameters and clinical severity within the SMA cohort.

A summary of the main corrected and exploratory SBM findings is provided in Table 2, which distinguishes the only finding that survived correction for multiple comparisons from nominal findings that did not remain significant after FDR adjustment.

### 3.4. Quantitative Associations

**Figure 1:**
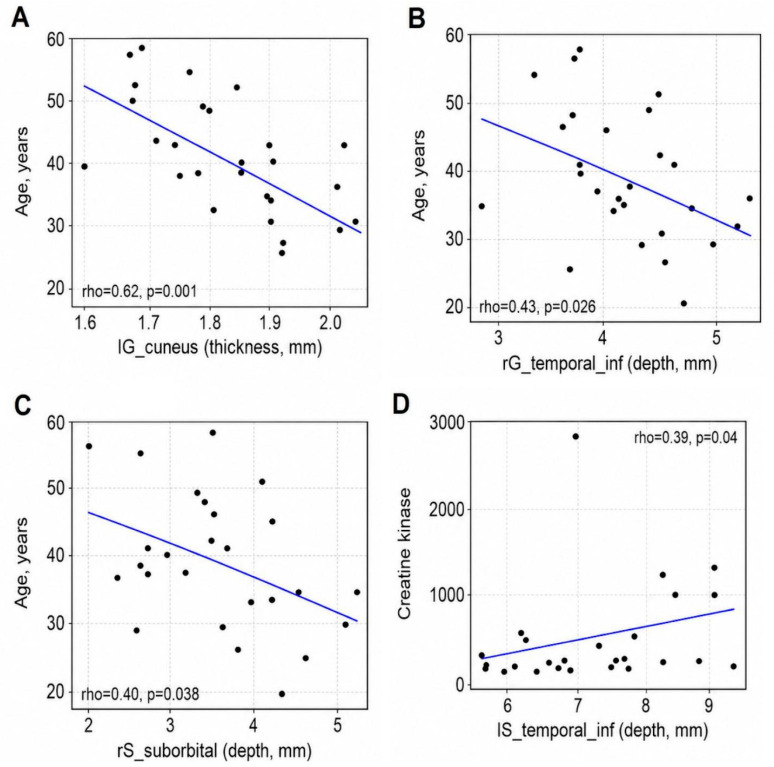
Relationships between selected surface-based morphometric parameters and age or creatine kinase level. The graphs show parameter pairs with significant results in Spearman correlation analysis. Panels (**A**–**C**) show associations between age and selected cortical or sulcal measurements: (**A**)—cortical thickness in the left cuneus; (**B**)—sulcal depth in the right inferior temporal region; and (**C**)—sulcal depth in the right suborbital sulcus. Panel (**D**) shows the relationship between creatine kinase level and sulcal depth in the left inferior temporal sulcus. Correlation coefficients and *p*-values are displayed within the panels.

Spearman correlation analysis revealed moderate negative correlations between age and selected surface-based morphometric parameters in the right suborbital sulcus, right inferior temporal gyrus, and left cuneus. In addition, a positive correlation was observed between creatine kinase level and sulcal depth of the left inferior temporal sulcus (Figure 1).

**Figure 2:**
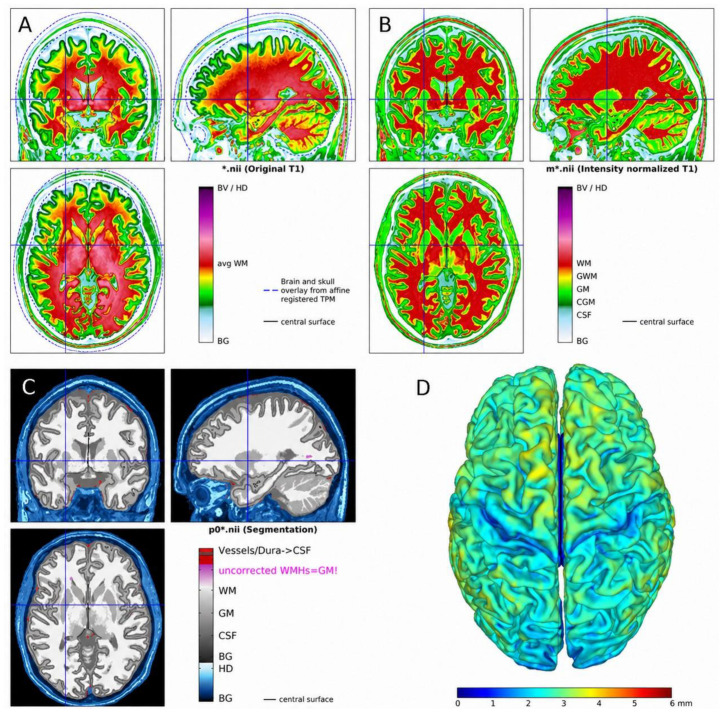
Representative CAT12 surface-based morphometry output with brain tissue segmentation. Panels (**A**,**B**) show grey matter and white matter tissue intensity maps before and after correction, displayed in axial, coronal, and sagittal planes. Panel (**C**) shows tissue segmentation into grey matter, white matter, and cerebrospinal fluid. Panel (**D**) presents a three-dimensional cortical surface reconstruction with a surface-based overlay illustrating cortical morphological variation. The figure is intended as a representative visualisation of image processing and segmentation quality rather than as a group-level statistical map.

Figure 2 presents an example visualisation of CAT12/SBM processing output for a single participant.

## 4. Discussion

### 4.1. Region-Based and Surface-Based Morphometry: Complementary Approaches

Advanced structural MRI techniques remain underused in SMA research. The present study addressed this gap by assessing cerebral morphological alterations in adults with SMA types 2 and 3 using CAT12-derived region-based morphometry and surface-based morphometry. We hypothesised that patients with SMA would show structural brain differences compared with healthy controls.

Region-based morphometry and surface-based morphometry provide complementary information about brain structure. CAT12-derived regional volumetric analysis enables assessment of grey matter volume within predefined atlas-based anatomical regions, including subcortical structures. In contrast, surface-based morphometry provides cortical measures such as cortical thickness, sulcal depth, gyrification index, and fractal dimension. Previous studies suggest that different morphometric approaches may vary in sensitivity depending on the neurological or psychiatric condition under investigation [23]. Combining regional volumetric and surface-based methods may therefore provide a broader characterization of cortical and subcortical brain morphology in SMA.

The main regional volumetric finding of the present study is lower grey matter volume in the left thalamus in adults with SMA compared with healthy controls. This result was derived from exported CAT12 regional volumetric values and should be interpreted as an atlas-defined regional finding. The finding is consistent with previous neuropathological and neuroimaging observations suggesting thalamic involvement in SMA, particularly in more severe phenotypes. The present results extend these observations by showing thalamic grey matter reduction in adult patients with SMA types 2 and 3 using in vivo structural MRI.

Although the left-sided localization of the thalamic finding is noteworthy, the present study was not designed to investigate hemispheric specialization or functional lateralization. Therefore, this structural alteration should primarily be interpreted as evidence of regional thalamic involvement rather than as a direct marker of language, memory, cognition, or other specific functional domains. Existing literature on thalamic asymmetry and thalamic vascular syndromes provides useful neuroanatomical context [24,25,26,27,28,29], but functional conclusions cannot be drawn from the present structural data alone.

The thalamus plays an important role in sensorimotor signal transmission and higher-order brain networks [26,27,28]. However, the present study did not include functional MRI, diffusion tractography, thalamic subnucleus segmentation, or detailed neuropsychological testing. Future multimodal studies are therefore required to determine whether the detected reduction in thalamic grey matter is associated with measurable functional consequences in adults with SMA.

### 4.2. Thalamic Involvement in SMA

Degenerative changes in the thalamus have previously been reported in SMA. Post-mortem studies have documented neuronal loss and neuronophagia in the thalamus and specific thalamic nuclei [9,30,31]. Thalamic degeneration has also been described together with cerebellar hypoplasia and cognitive impairment, particularly in infantile cases [30]. In addition, involvement of the striatum, cerebellum, and pons has been reported in severe SMA phenotypes, with degenerative changes associated with chromatolysis and neuronophagy.

Abnormalities in neurofilament regulation within chromatolytic thalamic neurons have also been described [32]. Because neurofilaments are produced in the neuronal cell body and undergo phosphorylation during axonal transport, abnormal neurofilament accumulation may suggest disturbed axonal transport or altered phosphorylation regulation. These mechanisms may contribute to neuronal vulnerability in SMA, although their direct relationship to the structural MRI findings observed in the present study remains uncertain.

Central nervous system abnormalities, particularly involving the thalami, have been suggested as possible early features of Werdnig–Hoffman disease [30]. However, attributing such findings exclusively to SMA requires caution, because hypoxia, malnutrition, severe systemic illness, or treatment-related factors may also contribute to brain abnormalities in severely affected patients [30,31]. Notably, thalamic changes have been reported on MRI in a child with SMA type 1 without documented hypoxic episodes, supporting the possibility of intrinsic degenerative processes [33].

Hayashi et al. [34] suggested that oxidative stress and altered glutamate transporter expression in SMA type 1 may contribute to latent thalamic degeneration. Although these mechanisms were described in severe infantile SMA, they provide a biologically plausible framework for considering thalamic vulnerability across the SMA spectrum.

In the present study, we considered potential confounding factors, including low Apgar scores, cortical abnormalities, hypoxic events, and limited early exposure to nusinersen in some patients. Given the short treatment exposure before MRI, the present findings should not be interpreted as reflecting long-term treatment-related effects. In contrast to some previous reports, we did not find significant grey matter volume changes in the motor cortex or cerebellum in adults with SMA types 2 and 3. Querin et al. [35] reported motor cortex enlargement in SMA type 4 compared with type 3, whereas Borba et al. [36] described cerebellar degeneration. The absence of corresponding findings in our cohort may reflect differences in SMA phenotype, disease severity, age, sample size, imaging methodology, or statistical approach.

Overall, our findings suggest selective thalamic involvement in living adults with SMA types 2 and 3 and are consistent with previous neuropathological observations. However, they should not be interpreted as evidence of a specific functional deficit or a definitive disease mechanism. The mechanisms underlying this alteration remain uncertain and may involve SMN-related neuronal vulnerability, developmental effects, neurodegenerative processes, or secondary network-level changes. Larger studies combining structural MRI, functional MRI, diffusion imaging, thalamic subnucleus analysis, and detailed clinical phenotyping are needed to validate this finding and clarify its clinical significance.

### 4.3. Surface-Based Morphometry Findings

Surface-based morphometry enables assessment of cortical parameters such as fractal dimension, cortical thickness, sulcal depth, and gyrification index [22,23].

In the present study, increased sulcal depth in the left orbital sulcus was the only cortical finding that remained statistically significant after correction for multiple comparisons. Other differences in cortical thickness and sulcal depth did not survive correction for multiple testing and should therefore be interpreted cautiously. Rather than representing established cortical abnormalities, these findings should be considered hypothesis-generating observations requiring validation in larger, independent cohorts.

Alterations in cortical thickness or sulcal depth may reflect multiple biological processes, including developmental variation, neurodegeneration, compensatory remodelling, or secondary network-level changes. Based on the present cross-sectional data, these mechanisms cannot be distinguished. Previous studies have also shown that sulcal depth may vary with ageing and other biological factors [22,37], further emphasising the need for longitudinal confirmation.

No significant group differences were found for gyrification index or fractal dimension. This suggests that, in the present cohort, these cortical complexity measures were not measurably altered in adults with SMA compared with healthy controls.

#### 4.3.1. Exploratory Associations with Sex and Age

Exploratory analyses suggested that selected morphometric parameters differed according to sex and age within the SMA group. Female patients showed shallower left and right orbital sulci than male patients, whereas participants younger than 40 years showed higher values in selected right orbital, right inferior temporal, and left cuneal regions. These findings should be interpreted cautiously because of the limited sample size and the exploratory nature of subgroup testing.

#### 4.3.2. Exploratory Associations with SMN2 Copy Number

Exploratory analyses suggested differences in cortical thickness and sulcal depth between healthy controls and SMA subgroups defined by SMN2 copy number. These observations may indicate a possible relationship between genetic background and regional brain morphology in SMA. However, because of the small subgroup sizes, they should be regarded as hypothesis-generating and not as definitive genotype–phenotype associations.

#### 4.3.3. Exploratory Associations with SMA Type

Subgroup analyses according to SMA type suggested differences in the left orbital sulcus between healthy controls and SMA subtypes. In the SMA type 3a subgroup, additional differences were observed in the left occipital pole and left cuneus. However, because only four participants had SMA type 2, the present study was not powered to detect robust subtype-specific brain alterations. These findings should therefore be interpreted as preliminary.

#### 4.3.4. Exploratory Associations with Motor Function

Analyses stratified by HFMSE score suggested differences between healthy controls and selected SMA subgroups in several cortical regions. However, these findings mainly reflected comparisons between controls and SMA subgroups rather than robust associations with clinical severity within the SMA cohort. They should therefore be interpreted as preliminary and require validation in larger cohorts with a broader range of motor impairment.

#### 4.3.5. Integrative Interpretation of Region-Based and Surface-Based Findings

The relationship between the thalamic region-based morphometry finding and the cortical surface-based observations remains uncertain. Although some cortical regions identified in exploratory analyses may be related to thalamic or associative networks, the present study cannot determine whether these alterations are structurally or functionally linked. Diffusion imaging and resting-state functional MRI would be required to assess structural and functional connectivity in future studies.

Overall, these preliminary findings support further investigation of central nervous system involvement in SMA using larger, longitudinal, and multimodal imaging studies.

## 5. Limitations

The present study has several limitations. First, the sample size was modest, and the SMA cohort was clinically heterogeneous, with only four participants having SMA type 2, limiting the reliability of subtype-specific analyses. Second, the cross-sectional design precluded assessment of disease progression or treatment-related effects. MRI examinations were performed either before treatment initiation or during the early loading phase of nusinersen therapy, with limited exposure before imaging; follow-up MRI examinations obtained after approximately one year of treatment were not included in the present analysis. Third, regional morphometric comparisons were performed without covariate adjustment for age, sex, or total intracranial volume. However, the SMA and control groups were well matched for these variables, and the findings should therefore be interpreted as unadjusted comparisons in demographically comparable cohorts. Finally, subgroup analyses were exploratory, cognitive data were limited to MoCA screening, and diffusion imaging, functional MRI, and thalamic subnucleus analysis were not performed, limiting functional and connectivity-related interpretation.

## 6. Conclusions

Adult patients with SMA types 2 and 3 showed reduced grey matter volume in the left thalamus and increased sulcal depth of the left orbital sulcus. Additional cortical differences identified by surface-based morphometry should be considered exploratory, as they did not remain significant after correction for multiple comparisons. These findings are consistent with the concept that SMA may extend beyond lower motor neuron degeneration and involve selected brain structures. Larger longitudinal and multimodal studies are needed to validate these observations and clarify their clinical significance.

## Figures and Tables

**Table 1:** Detailed characteristics of the study group according to the SMA subtype.

Variable	SMA Type 2 (*n* = 4)		SMA Type 3a (*n* = 12)		SMA Type 3b (*n* = 11)	
	M ± SD/*n* (%)	Range	M ± SD/*n* (%)	Range	M ± SD/*n* (%)	Range
Age, years	34.47 ± 3.20	31.78–39.00	36.10 ± 11.98	19.35–56.45	39.56 ± 7.61	25.07–52.37
**Gender**						
Female	2 (50.0)	-	7 (58.3)	-	6 (54.5)	-
Male	2 (50.0)	-	5 (41.7)	-	5 (45.5)	-
**No of SMN2 gene copies**						
3	4 (100.0)	-	6 (50.0)	-	6 (54.5)	-
4	0 (0.0)	-	6 (50.0)	-	5 (45.5)	-
First symptoms, years	0.75 ± 0.54	0.20–1.50	2.18 ± 0.69	1.00–3.00	10.55 ± 5.68	4.00–22.00
Confirmed diagnosis, years	2.00 ± 0.00	2.00–2.00	8.08 ± 14.56	2.00–54.00	20.00 ± 12.46	5.00–44.00
Chop-Intend *	13.25 ± 11.56	1.00–27.00	37.11 ± 11.41	17.00–51.00	45.50 ± 0.71	45.00–46.00
HFMSE	1.00 ± 1.15	0.00–2.00	15.17 ± 13.86	3.00–47.00	37.55 ± 16.44	8.00–61.00
**HFMSE**						
≤10	4 (100.0)	-	7 (58.3)	-	1 (9.1)	-
>10	0 (0.0)	-	5 (41.7)	-	10 (90.9)	-
**Motor milestones**						
Achieved independent sitting	4 (100.0)	-	12 (100.0)	-	11 (100.0)	-
Achieved independent walking	0 (0.0)	-	12 (100.0)	-	10 (90.9)	-
**Current state**						
Sits	3 (75.0)	-	9 (75.0)	-	2 (18.2)	-
Walks	0 (0.0)	-	3 (25.0)	-	9 (81.8)	-
Lying in bed	1 (25.0)	-	0 (0.0)	-	0 (0.0)	-
Creatine kinase	45.25 ± 22.08	14.00–66.00	207.25 ± 307.27	19.00–1157.00	652.45 ± 803.91	58.00–2860.00
**Creatine kinase**						
<171	4 (100.0)	-	9 (75.0)	-	4 (36.4)	-
171–855	0 (0.0)	-	2 (16.7)	-	4 (36.4)	-
855–1710	0 (0.0)	-	1 (8.3)	-	2 (18.2)	-
>1710	0 (0.0)	-	0 (0.0)	-	1 (9.1)	-
**Comorbidities**						
Advanced scoliosis	4 (100.0)	-	4 (33.3)	-	2 (18.2)	-
Mild/moderate scoliosis	0 (0.0)	-	1 (8.3)	-	0 (0.0)	-
Scoliosis surgery	0 (0.0)	-	5 (417)	-	0 (0.0)	-
Nephrolithiasis	1 (25.0)	-	0 (0.0)	-	1 (9.1)	-
History of cholelithiasis/cholecystectomy	1 (25.0)	-	1 (8.3)	-	1 (9.1)	-
Chronic respiratory failure	1 (25.0)	-	1 (8.3)	-	0 (0.0)	-
Arterial hypertension	0 (0.0)	-	1 (8.3)	-	2 (18.2)	-
Type 2 diabetes mellitus/insulin resistance	0 (0.0)	-	2 (16.7)	-	0 (0.0)	-
Cardiac arrhythmias	0 (0.0)	-	2 (16.7)	-	0 (0.0)	-
Overweight/obesity (BMI > 25 kg/m^2^)	0 (0.0)	-	2 (16.7)	-	2 (18.2)	-
Underweight (BMI < 18.5 kg/m^2^)	2 (50.0)	-	2 (16.7)	-	1 (9.1)	-

M—mean, SD—standard deviation, * only for non-ambulatory patients (*n* = 4 for SMA type 2, *n* = 9 for SMA type 3a, *n* = 2 for SMA type 3b).

**Table 2:** Comparison of cortical depth (mm) between the study group and the control group in selected locations.

Location	Study Group (*n =* 27)	Control Group (*n =* 27)	MD (95% CI)	*p*	*p′*
rG_front_middle	4.11 ± 0.58	3.75 ± 0.39	0.36 (0.09; 0.63)	**0.010**	0.386
lG_rectus	3.14 ± 0.54	3.61 ± 0.57	−0.47 (−0.78; −0.17)	**0.003**	0.225 ***
rG_temporal_inf	4.30 (3.81; 4.72)	4.69 (4.20; 5.65)	−0.39 (−0.94; −0.08)	**0.019**	0.400
lS_orbital-H_Shaped	12.69 ± 0.97	11.64 ± 0.80	1.04 (0.56; 1.53)	**<0.001**	0.012 *
rS_orbital-H_Shaped	12.48 ± 0.92	11.82 ± 0.70	0.66 (0.21; 1.11)	**0.005**	0.226 ***
rS_suborbital	3.55 ± 0.92	2.89 ± 1.02	0.66 (0.13; 1.19)	**0.016**	0.400
lS_temporal_inf	7.19 ± 1.02	7.94 ± 1.21	−0.76 (−1.37; −0.15)	**0.016**	0.400

Data are presented as mean ± standard deviation or median (interquartile range), depending on the normality of the distribution. MD—mean or median difference (study group vs. control group), CI—confidence interval. The *p*-value was calculated using Student’s *t*-tests, Welch’s *t*-tests, or Mann–Whitney U tests, depending on the fulfillment of the conditions of distribution normality and homogeneity of variances. The *p*′ value was calculated using the Benjamini–Hochberg correction for multiple comparisons (* *p*′ < 0.05, *** *p*′ < 0.25), rG_front_middle—right middle frontal gyrus, lG_rectus—left straight gyrus, rG_temporal_inf—right inferior temporal gyrus, lS_orbital-H_Shaped—left H-shaped orbital sulcus, rS_orbital-H_Shaped—right H-shaped orbital sulcus, rS_suborbital—right suborbital sulcus, lS_temporal_inf—left inferior temporal sulcus. The statistically significant changes are highlighted in bold.

## Data Availability

The raw data supporting the conclusions of this article will be made available by the authors on reasonable request, subject to ethics approval and privacy restrictions.
